# Complanatuside alleviates inflammatory cell damage induced by pro-inflammatory cytokines in skin keratinocytes

**DOI:** 10.3389/fchem.2022.909651

**Published:** 2022-08-10

**Authors:** Xiao Wang, Xuetao Xu, Panpan Wu, Mengshuo Wu, Lishe Gan, Jingwei Jin, Rihui Wu, Wenfeng Liu, Kun Zhang, Dongli Li, Xi Zheng

**Affiliations:** ^1^ School of Biotechnology and Health Sciences, Wuyi University, Jiangmen City, China; ^2^ International Healthcare Innovation Institute (Jiangmen), Jiangmen, China; ^3^ Department of Chemical Biology, Ernest Mario School of Pharmacy, Rutgers, the State University of New Jersey, Piscataway, NJ, United States

**Keywords:** HaCaT cells, skin inflammatory damage, complanatuside, pyroptosis, COVID-19

## Abstract

Cytokine-mediated inflammatory response is considered a cause of skin lesion in COVID-19 patients. Complanatuside is a flavonol glycoside isolated from *Astragalus complanatus*. Flavonoids from *Astragalus complanatus* were reported to have anti-inflammatory and anticancer activities but the potential protective effect of complanatuside on cytokine-induced inflammatory damage in skin keratinocytes is not known. The aim of this study is to explore the inhibitory effect of complanatuside on inflammation and its underlying mechanisms in skin epithelial HaCaT cells treated with inflammatory cytokines. The combination of IFN-γ, TNF-α, and IL-6 decreased cell viability, increased cell death, and pyroptosis in HaCaT cells. Treatment with complanatuside alleviated the effects of the cytokine combination on HaCaT cells. Complanatuside down-regulated pyroptosis related to NLRP3, GSDMD, and ASC. The effects of complanatuside were related to vast reductions in the levels of iNOS, COX-2, and ROS. Results of the present study indicate that complanatuside inhibited inflammation and protected the cells from inflammatory cell damage in HaCaT cells treated with the combination of IFN-γ, TNF-α, and IL-6. Complanatuside may be a promising candidate for inhibiting COVID-19 related skin inflammatory damage.

## Graphical Abstract

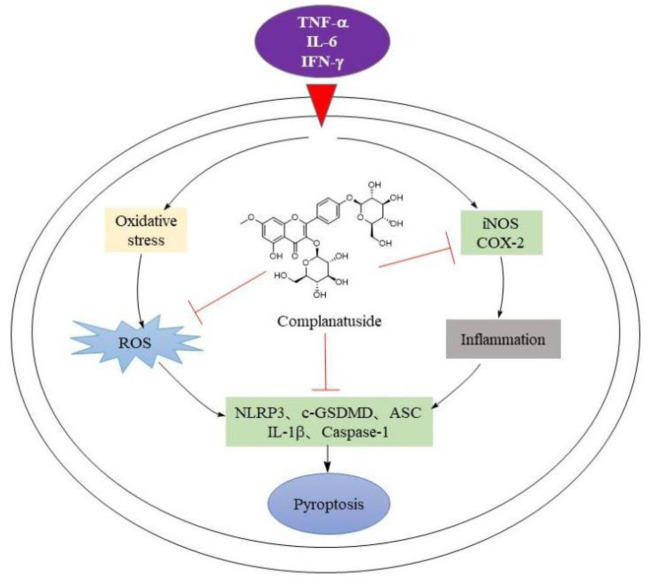

## Introduction

Severe acute respiratory syndrome coronavirus 2 (SARS-CoV-2) is the cause of Coronavirus disease-19 (COVID-19), which is a worldwide epidemic (Huang et al., 2020). The COVID-19 pandemic has affected many countries and caused an overwhelming of health care systems and both psychological and economic burdens. Although COVID-19 is best known to cause interstitial pneumonia and respiratory failure (Novak et al., 2021), skin manifestations as extrapulmonary signs associated with COVID-19 have been increasingly reported (Marzano et al., 2020; Matar et al., 2020). According to the recent classification, as shown by a study from Spain, the most typical presentations of skin lesions include chilblain-like lesions, ischemic-livedoid/necrotic lesions, and varicelliform-like/vesicular eruptions. Vacuolar interface dermatitis with scattered dead keratinocytes has been found in chilblain-like lesions. Histopathology of ischemic-livedoid/necrotic lesions related to COVID-19 is characterized by epidermal necrosis and thrombotic vascular lesions (Rongioletti et al., 2021). The COVID-19 skin lesions are plausibly associated with the excessive inflammation response of the patients characterized by pathological cytokine levels. However, the pathogenesis of the skin lesions in COVID-19 patients is not clear. It has been hypothesized that the increase in COVID-19 related release of interferon and inflammatory cell damage induced by cytokines may cause the skin lesions (Grimm et al., 2021).

Coronavirus, especially SARS-CoV-2, can cause a “cytokine storm” by increasing the production and release of pro-inflammatory cytokines (Barnes et al., 2020). Studies have shown that the cytokine storm is directly related to lung injury, multiple organ failure, and death of COVID-19 (Gao et al., 2020; Sun et al., 2020). TNF-α, IL-6, IFN-γ, IL-18, IL-15, IL-1α, IL-1β, and IL-2 are among the most highly upregulated pro-inflammatory cytokines in COVID-19 (Lucas et al., 2020; Karki et al., 2021).

Studies indicate that IL-6, TNF-α, and IFN-γ has become a significant feature of COVID-19 patient deterioration (Fara et al., 2020; Gubernatorova et al., 2020). Inflammatory cell death may be a possible mechanistic link between cytokine storm and organ damage. A recent study showed that among the eight cytokines tested, TNF-α in combination with IFN-γ strongly induced inflammatory cell death, and administration of this cytokine combination to mice resulted in lethal cytokine shock which resembles COVID-19 related tissue damage and inflammation (Karki et al., 2021).

Results of this study indicated that pathological levels of multiple cytokines can cause inflammatory cell death and contribute to tissue damage in COVID-19 patients. It is of great interest to investigate the effects of multiple cytokines on skin keratinocytes and explore the possible mechanistic link between cytokine storm and skin lesions in COVID-19 patients.

**FIGURE 1:**
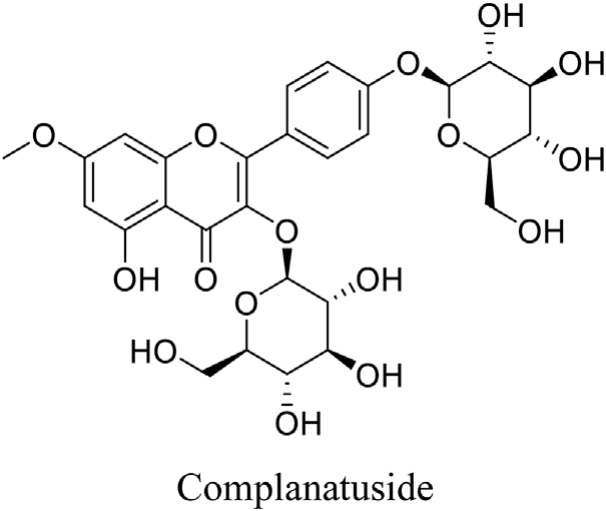
Structure of complanatuside.

Complanatuside is a flavonol glycoside (Figure 1) present in *Astragalus complanatus*. That is commonly used as a traditional Chinese medicine in clinics. Flavonoids from *Astragalus complanatus* have been reported to exert inhibitory effects on inflammation and cancer (Ng et al., 2014; Yao et al., 2018). In this study, IL-6, TNF-α, and IFN-γ were used to simulate “cytokine storm” to induce inflammatory injury in HaCaT cells. We utilized this model to determine the inhibitory effect of complanatuside on inflammation and explore the mechanisms underlying its actions.

## Materials and methods

### Cell line and cell culture

HaCaT cells were obtained from the American Type Culture Collection (ATCC, Rockville, MD, United States ). Dulbecco’s Modified Eagle Medium (DMEM) medium supplemented with 10% fetal bovine serum (FBS), penicillin (100 units/mL), and streptomycin (100 mg/ml) (all from Gibco, Grand Island, NY, United States ) was used for the culture of HaCaT cells. The cells were grown at 37°C in a humidified atmosphere of 5% CO_2_. IFN-γ was purchased from Beijing T&L Biotechnology Co., Ltd. TNF-α and IL-6 were obtained from Shanghai Bohu Biotech Co., Ltd. Complanatuside was obtained from Meilunbio (Dalian, China). The compounds were dissolved in dimethyl sulfoxide (DMSO, Sigma-Aldrich, St. Louis, MO, United States ) as a stock solution and stored at −20°C and the final concentration of DMSO in all experiments was 0.2%.

### Cell viability assay

HaCaT cells were grown in 96-well plates at a cell density of 5 × 10^3^ cells/well and incubated for 24 h to allow the attachment of the cells to the culture plates. The cells were treated with serum-free DMEM medium for 24 h, then treated with various concentrations of complanatuside and incubated in the absence or presence of cytokine combinations (80 ng/ml IFN-γ, 40 ng/ml TNF-α, and 20 ng/ml IL-6) for 24 h. At the end, MTT solution (200 μl; 0.5 mg/ml in DMEM medium without serum) was added to each of the wells. The plate was incubated at 37°C for 4 h. After carefully removing the medium, 200 µl DMSO was added to each well. The absorbance at 570 nm was measured by a microplate reader.

### Trypan blue exclusion assay

HaCaT cells (5 ×10^5^ cells/mL) were grown in 35-mm cell culture dishes (2 ml/dish) and incubated in DMEM +10% FBS for 24 h. The cells were treated with serum-free DMEM medium for 24 h, the media were subsequently changed to different concentrations of complanatuside with a cytokine combination of IFN-γ (80 ng/ml), TNF-α (40 ng/ml), and IL-6 (20 ng/ml) alone or in cytokine combination for 24 h. After treatment, the cells were digested by trypsin to make a single cell suspension. 10 µl of cell suspension was mixed with 10 µl of 0.4% trypan blue solution for 2 min. The number of viable and dead cells was counted by the countess automatic cell counter (Countess^®^, AMQAX1000, Thermo, United States ).

### ROS measurement

ROS were examined using 2′,7′-dichlorfluoroescein diacetate (DCFH-DA) and ROS ELISA Kit (Shanghai Enzyme-linked Biotech Co., Ltd, China). HaCaT cells (1 × 10^6^ cells/well) were plated in 35-mm cell culture dishes for 24 h. After HaCaT cells were treated with cytokine combinations and complanatuside, the supernatants were gathered and subjected to the analysis of ROS by using the ROS ELISA Kit. After washing with PBS buffer, cells were incubated with 10 μM DCFH-DA in the culture medium at 37°C for 30 min in the dark. After washing, the fluorescent signals (excitation at 488 nm and emission at 525 nm) in the cells were determined by a fluorescence microplate reader.

### IL-1β assay

To determine IL-1β, HaCaT cells (1 × 10^6^ cells/well) were plated in 6-well plates and incubated for 24 h to allow the attachment of cells to the culture plates. The cells were treated with serum-free DMEM medium for 24 h, then treated with various concentrations of complanatuside and incubated in the absence or presence of cytokine combination (80 ng/ml IFN-γ, 40 ng/ml TNF-α, and 20 ng/ml IL-6) for 24 h. After treatment, the culture supernatants were gathered and subjected to the analysis of IL-1β by using the IL-1β ELISA Kit (ab214025).

### Caspase-1 assay

Caspase-1 fluorescence staining was done using the Caspase-1 (active) Staining Kit-Green Fluorescence (#ab219935, Abcam Ltd., Waltham, MA). HaCaT cells were treated with various concentrations of complanatuside in the presence or absence of cytokine combination (80 ng/ml IFN-γ, 40 ng/ml TNF-α, and 20 ng/ml IL-6). After treatment, FAM-YVAD-FMK was added into the cell solution and incubated for 1 h at 37°C. Caspase-1 staining was analyzed in cells by a fluorescence microscope. For the caspase-1 activity assay, the Caspase-1 assay kit (Fluorometric) (#ab39412, Abcam) was used. In brief, cell lysates were prepared after treatment. Reaction buffer and YVAD-AFC substrate were mixed with cell lysates and incubated for 1 h at 37°C. Samples were analyzed using a fluorescence microplate reader at an excitation wavelength of 400 nm and an emission wavelength of 505 nm.

### Western blot

At the end of the experiment, the cells were lysed to prepare cell lysates. Proteins were loaded and separated by sodium dodecyl sulfate polyacrylamide gel electrophoresis (SDS-PAGE) and transferred to a nitrocellulose membrane. After blocking nonspecific binding sites with blocking buffer, the membranes were incubated overnight at 4°C with primary antibodies. β-Actin (#8457, CST) was used as a loading control. After the removal of the primary antibodies (COX-2, #12282, CST; iNOS, AF0199, Affinity; ASC, #13833, CST; GSDMD, AF4012, Affinity; NLRP3, AG-20B-0014-C100, AdipoGen), the membranes were then washed three times with TBST buffer (100 mM NaCl, 10 mM Tris-HCl, pH 7.5, and 0.1% Tween-20) at room temperature and incubated with secondary antibody. Next, the membrane was washed with TBST buffer three times. Finally, protein bands were detected using the Pierce™ ECL Western Blotting Substrate (Thermo Scientific, United States ).

### Statistical analysis

Statistical analyses were done by using the software Graphpad Prism. All data were presented as mean ± SD. One-way ANOVA was performed to evaluate the difference between groups. The differences were considered statistically significant at *p* < 0.05.

## Results

### Effect of IFN-γ, TNF-α and IL-6 on the viability of HaCaT cells

**FIGURE 2:**
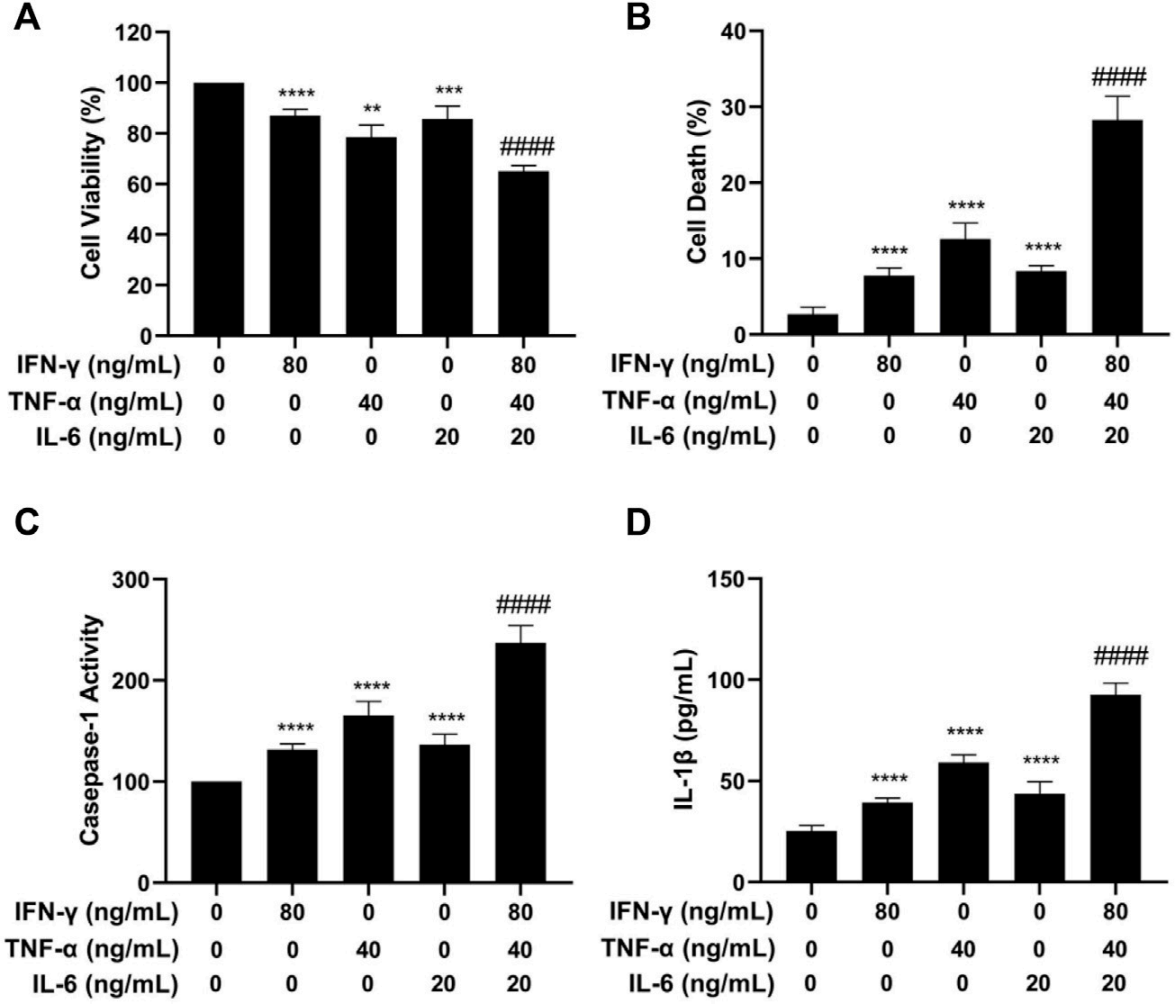
Effects of IFN-γ, TNF-α, and IL-6 on cell viability, cell death, caspase-1 activation, and IL-1β level of HaCaT cells. HaCaT cells were exposed to IFN-γ, TNF-α, and IL-6 alone or in combination for 24 h. **(A)** The MTT assay was used to determine the cell viability. **(B)** The Trypan blue exclusion assay was used to determine cell death. **(C)** Caspase-1 activity was detected by the caspase-1 assay kit. **(D)** IL-1β level was measured by ELISA. The data were presented as mean ± SD (*n* = 3), ** = *p* < 0.01, *** = *p* < 0.001, and **** = *p* < 0.0001 as compared with the cells treated with IFN-γ, TNF-α, or IL-6 alone.

Initial experiments were carried out by treating HaCaT cells with cytokines of IFN-γ, TNF-α, and IL-6 alone or in combination. After a 24 h-treatment, the MTT assay was used to determine cell viability. As shown in Figure 2A, after treatment with IFN-γ, TNF-α, and IL-6 separately or in combination, the average survival rate of HaCaT cells was 87.1%, 78.1%, 85.6%, and 65.1%, respectively. The combined effect of IL-6, TNF-α, and IFN-γ on cell viability was more obvious than that of single factor alone, and the survival rate was obviously lower in the combination-treated cells than that in the single cytokine-treated cells. The result of the study described above indicated that the combination of IFN-γ, TNF-α, and IL-6 strongly decreased the HaCaT cell viability.

### IFN-γ, TNF-α, and IL-6 induce cell death in HaCaT cells

We next investigated the effect of the cytokines separately or in combination on HaCaT cell death using the trypan blue exclusion assay. As shown in Figure 2B, the combination of IFN-γ, TNF-α, and IL-6 strongly increased the number of dead cells. The combination of IFN-γ, TNF-α, and IL-6 had a more potent effect on inducing cell death than each cytokine used alone (*p* < 0.0001). To further investigate if the cytokine combination induced pyroptosis, caspase-1 activation and IL-1β were examined in HaCaT cells after treatment with IFN-γ, TNF-α, and IL-6. Caspase-1 activation contributes to the maturation of cytokines and membrane pore forming protein gasdermine during the process of pyroptosis and can be used as a marker of pyroptosis. As displayed in Figure 2C, treatment of HaCaT cells with the cytokine combination strongly increased the activity of caspase-1. Statistical analysis showed that the cells treated with the cytokine combination had a significantly higher level of caspase-1 activity (*p* < 0.0001) as compared to the cells treated with a single cytokine.

IL-1β is a proinflammatory cytokine that is released by the host cells and is involved in the process of pyroptosis. The effects of IFN-γ, TNF-α, and IL-6 alone or in combination on increasing the levels of IL-1β were determined using the ELISA assay. As illustrated in Figure 2D, treatment of HaCaT cells with the cytokine combination strongly increased the level of IL-1β. The differences in the level of IL-1β between the single cytokine-treated group and the cytokine combination-treated group were statistically significant (*p* < 0.0001). The results of caspase-1 and IL-1β experiments indicated that the IFN-γ, TNF-α, and IL-6 in combination induced pyroptosis in the HaCaT cells.

### Complanatuside alleviates the effect of IFN-γ, TNF-α, and IL-6 on decreasing cell viability

**FIGURE 3:**
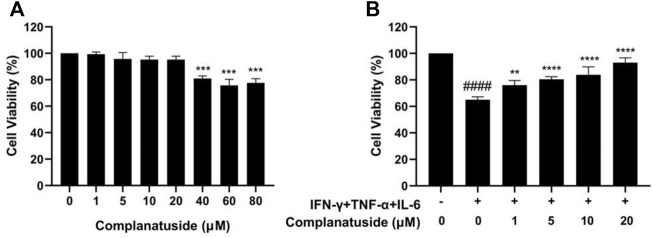
Effects of complanatuside on the viability of HaCaT cells with or without the cytokine combination treatment. **(A)** HaCaT cells were treated with various concentrations of complanatuside for 24 h. The MTT assay was used to determine cell viability. The results were shown as mean ± SD (*n* = 3), *** = *p* < 0.001 as compared with control cells. **(B)** HaCaT cells were treated with various concentrations of complanatuside in the absence or presence of cytokine combination and the cell viability was determined by MTT assay. Mean ± SD from three separate experiments were shown. **####** = *p* < 0.0001 as compared with control group, *** = *p* < 0.001, and **** = *p* < 0.0001 as compared with cells treated with cytokine combination.

Cytotoxicity of complanatuside was evaluated using the MTT assay before the evaluation of its potential protective effect on cytokine-induced cell damage in HaCaT cells. In these experiments, HaCaT cells were treated with different concentrations of complanatuside for 24 h, and the cell viability was measured using the MTT assay. As shown in Figure 3A, complanatuside did not alter the cell viability significantly at concentrations up to 20 µM. Statistical analysis exhibited that the differences in cell viability between the control group and different concentrations of complanatuside-treated groups (1, 5, 10, and 20 μM) were not statistically significant (*p* > 0.05). Higher concentrations of complanatuside (40, 60, and 80 μM) significantly reduced the cell viability (*p* < 0.0001). The results indicated that complanatuside had no toxicity to the skin keratinocytes at a concentration range of 1–20 μM. Therefore, all subsequent experiments were conducted using these non-toxic concentrations.

As shown in Figure 3B, treatment with the combination of IFN-γ, TNF-α, and IL-6 significantly decreased the viability of HaCaT cells. It was noticed that complanatuside increased the cell viability of HaCaT cells treated with the cytokine combination. When HaCaT cells were treated with complanatuside at 1, 5, 10, and 20 μM, the cell survival rates as compared to the cytokine combination-treated cells were increased by 11.0, 15.3, 18.7, and 28.0%, respectively. Results of our study suggested that complanatuside at non-toxic concentrations protected HaCaT cells from the cytokine combination-induced decrease in cell viability.

### Protective effect of complanatuside on HaCaT cell death induced by TNF-α, IFN-γ, and IL-6

**FIGURE 4:**
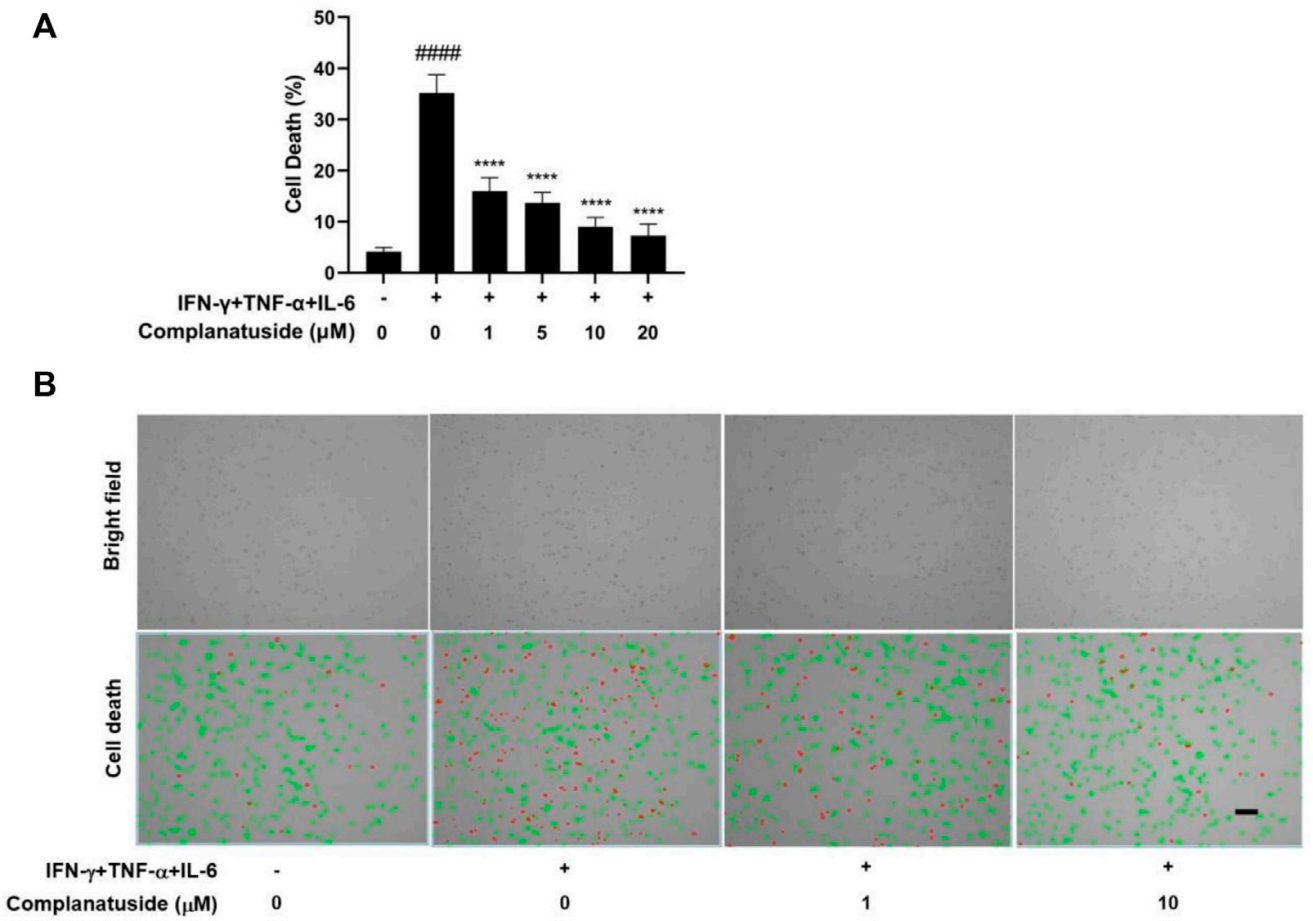
Protective effect of complanatuside on HaCaT cell death induced by the cytokine combination. HaCaT cells were treated with various concentrations of complanatuside in the absence or presence of cytokine combination and cell death was determined by the Trypan blue exclusion assay. **(A)** Percentage of cell death in HaCaT cells treated with the cytokine combination in the presence or absence of complanatuside. The data were presented as mean ± SD (*n* = 3), **####** = *p* < 0.0001 as compared with the control group, **** = *p* < 0.0001 as compared with the cytokine combination-treated group. **(B)** Images of control cells, cytokine combination-treated cells, cells treated with cytokine combination, and complanatuside (1 or 10 μM) were taken by the Countess^®^ automatic cell counter. Red color represents dead cells and green represents living cells (Size bar = 50 µm).

The potential protective effect of complanatuside on HaCaT cell death induced by the combination of TNF-α, IFN-γ, and IL-6 was examined using the trypan blue exclusion assay and the result was shown in Figure 4. The cytokine combination strongly induced cell death in HaCaT cells (Figure 4A). Treatment with complanatuside reduced the number of dead cells. Complanatuside at 1, 5, 10, and 20 μM decreased the dead cells by 19.2, 21.5, 26.2, and 27.9%, respectively. Representative graphs of the trypan blue assay by the Countess II cell counter were shown in Figure 4B. Cells labeled with green are alive cells, and those labeled red are dead cells. As illustrated in Figure 4B, the number of red cells increased in cytokine-induced cells as compared with the negative control. Treatment with complanatuside markedly decreased the red cells, indicating that complanatuside could protect HaCaT cells from cytokine-induced cell death in HaCaT cells.

**FIGURE 5:**
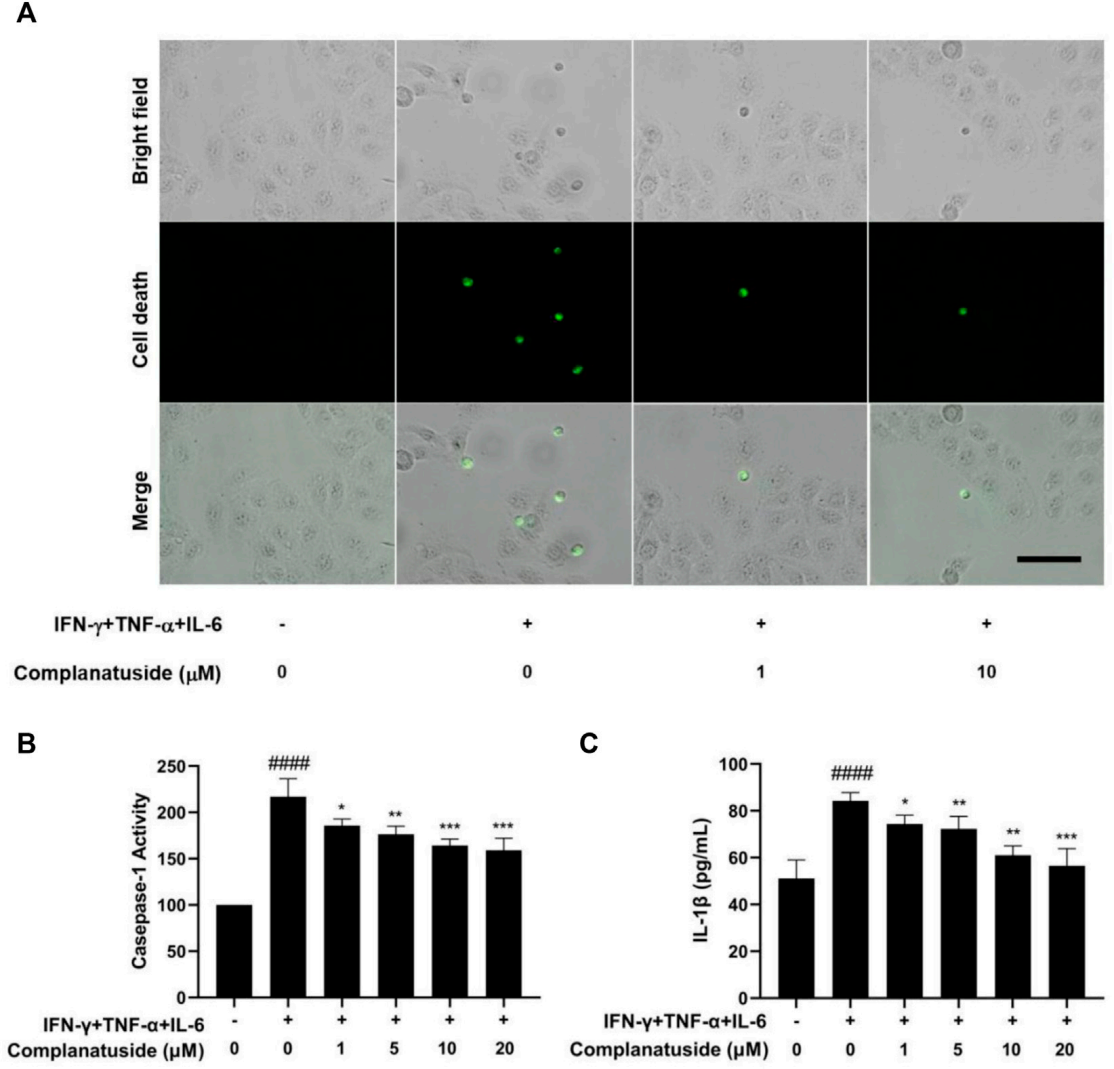
Protective effect of complanatuside on pyroptosis in HaCaT cells induced by the cytokine combination. **(A)** Representative micrographs of caspase-1 staining in HaCaT cells treated with complanatuside in the presence or absence of the cytokine combination (Size bar = 25 µm). **(B)** Caspase-1 activity in HaCaT cells was measured using the caspase-1 assay kit. **(C)** ELISA was used to measure the levels of IL-1β. The data were presented as mean ± SD (*n* = 3), **####** = *p* < 0.0001 as compared with control group, * = *p* < 0.05, ** = *p* < 0.01, and *** = *p* < 0.001 as compared with the cells treated with cytokine combination.

We further explored the effect of complanatuside on cytokine-induced inflammatory cell death, pyroptosis, in HaCaT cells. Figure 5 showed the results of pyroptosis in HaCaT cells as indicated by the increase in caspase-1 and IL-1β. Treatment of the cells with complanatuside decreased the activation of caspase-1 induced by the cytokine combination. Representative micrographs of caspase-1 fluorescence staining in HaCaT cells are shown in Figure 5A. More caspase-1 positive cells were found in HaCaT cells treated with the cytokine combination as compared with the negative control. Treatment with complanatuside decreased the number of caspase-1 positive cells (Figure 5A). Caspase-1 was also tested using the caspase-1 activity assay kit. As shown in Figure 5B, treatment of the cells with complanatuside significantly decreased the caspase-1 activity induced by the cytokine combination (*p* < 0.001). ELISA was used to determine the effect of complanatuside on the levels of IL-1β in HaCaT cells. As shown in Figure 5C, treatment with complanatuside reduced the elevated level of IL-1β caused by the cytokine combination. Treatment with complanatuside at 1, 5, 10, and 20 μM decreased the levels of IL-1β by 11.7, 14.2, 27.6, and 32.9%, respectively. The results of these experiments indicated that complanatuside strongly alleviated pyroptosis induced by the combination of TNF-α, IFN-γ, and IL-6 in HaCaT cells.

### Effects of complanatuside on NLRP3 GSDMD ASC

**FIGURE 6:**
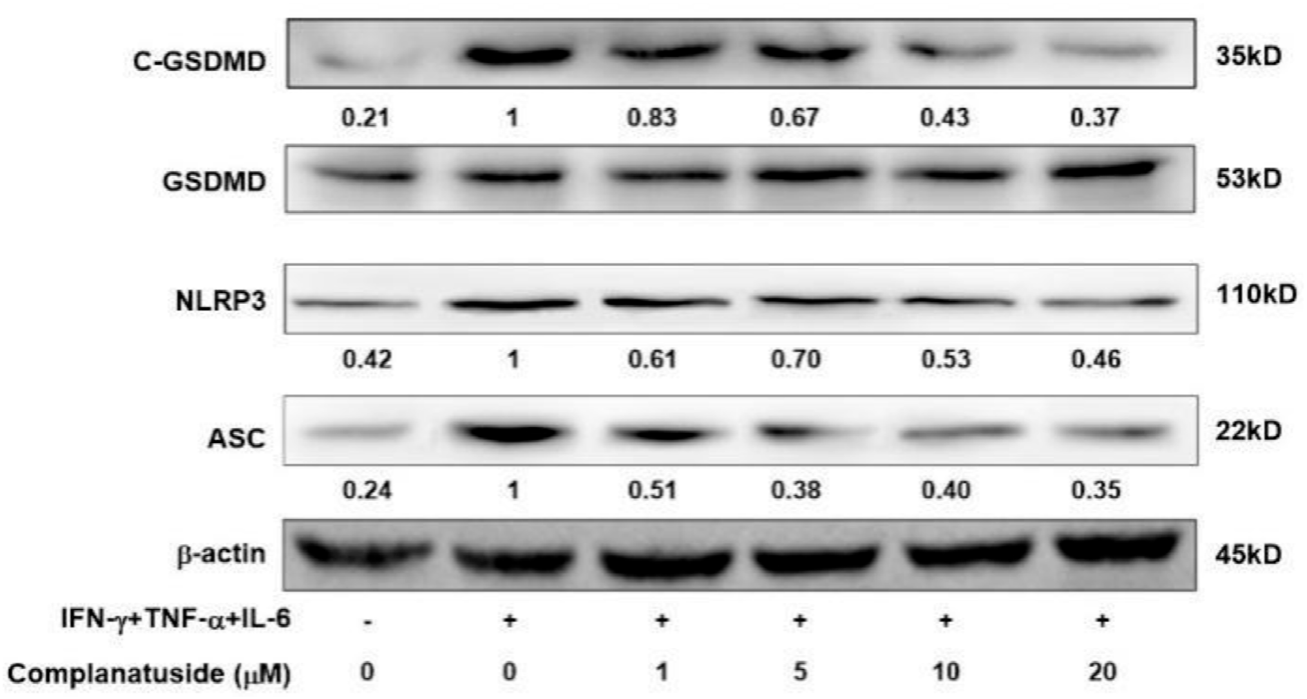
Effects of complanatuside on the levels of NLRP3, ASC, and cleaved GSDMD in HaCaT cells treated with the cytokine combination. HaCaT cells were treated with various concentrations of complanatuside with or without co-treatment with the cytokine combination for 24 h. The levels of NLRP3, ASC, and cleaved GSDMD were determined by Western blot analysis.

Studies have shown that pyroptosis in COVID-19 may be associated with activation of the NLRP3 associated pyroptosis pathway (Song et al., 2020). Therefore, we measured the levels of NLRP3, ASC, and GSDMD in HaCaT cells. As shown in Figure 6, the levels of NLRP3, ASC, and cleaved GSDMD markedly increased in the cells exposed to the cytokine combination. Treatments with complanatuside strongly inhibited cytokine combination-induced NLRP3, ASC, and cleaved GSDMD expression, indicating that complanatuside had a potent inhibitory effect on the activation of pyroptosis pathway in HaCaT cells exposed to the combination of TNF-α, IFN-γ, and IL-6.

### Effects of complanatuside on cytokines combination-induced iNOS, COX-2 and ROS

**FIGURE 7:**
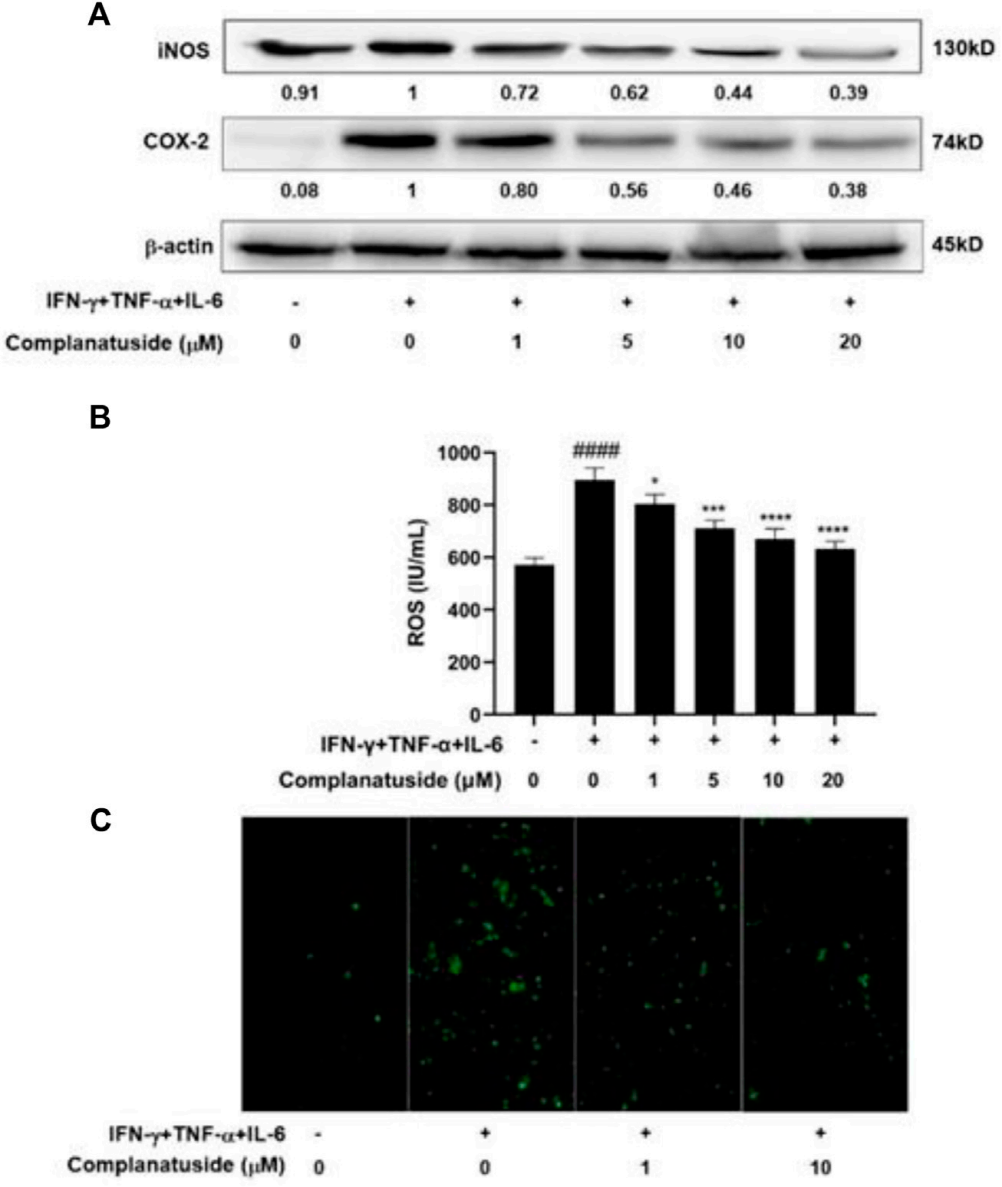
Effects of complanatuside on the levels of iNOS, COX-2, and ROS in HaCaT cells treated with the cytokine combination. HaCaT cells were treated with various concentrations of complanatuside with or without co-treatment with the cytokine combination for 24 h. **(A)** The levels of iNOS and COX-2 were determined by Western blot analysis. **(B)** ROS was detected using the fluorescence dye DCFH-DA and ELISA. ROS levels in HaCaT cells treated with complanatuside with or without co-treatment with the cytokine combination. The data were presented as mean ± SD. Asterisk indicates comparison between the cytokine combination-treated group and complanatuside-treated groups (* = *p* < 0.05, *** = *p* < 0.001, and **** = *p* < 0.0001). The number sign # indicates comparison between the cells treated with cytokine combination and the cells in control group (**####** = *p* < 0.0001). **(C)** Representative micrographs of ROS fluorescence staining of HaCaT cells treated with complanatuside in the presence or absence of the cytokine combination (Size bar = 50 µm).

To further explore the mechanisms for inhibition of the NLRP3 pathway by complanatuside, we determined its effect on iNOS and COX-2, which have been shown to be associated with the activation of NLRP3 inflammasome (Kundu et al., 2018; Cinelli et al., 2020; Liao et al., 2021). As shown in Figure 7A, the cytokine combination strongly increased the levels of iNOS and COX-2 in HaCaT cells. Treatment of the cells with different concentrations of complanatuside dose-dependently reduced the elevated levels of iNOS and COX-2 in HaCaT cells induced by the cytokine combination. The results of these experiments suggested that inhibition of iNOS and COX-2 was involved in mediating the effect of complanatuside in cytokine-induced pyroptosis.

In additional experiments, we investigated the effects of cytokine combination and complanatuside on the levels of ROS in HaCaT cells. As shown in Figure 7B, the cytokine combination induced a distinct increase in the level of ROS, and treatment with complanatuside dose-dependently decreased the elevated level of ROS induced by the cytokine combination. The average inhibition rates in the cells treated with 1, 5, 10, and 20 μM of complanatuside were 10.1, 20.6, 25.1, and 29.5%, respectively. Representative micrographs of ROS fluorescence staining in the cells are shown in Figure 7C. The fluorescence intensity of ROS in HaCaT cells treated with the cytokine combination was visibly increased as compared with that in the control group, and treatment with complanatuside reduced the fluorescence intensity in the cells. The result of this study indicated that inhibition of inflammatory cell death by complanatuside in HaCaT cells was associated with strong suppression of ROS.

## Discussion

According to current research, we demonstrated that the combination of IFN-γ, TNF-α, and IL-6 strongly decreased the viability of HaCaT skin keratinocytes. This cytokine combination also strongly increased the death of HaCaT cell. Our study further indicated that the HaCaT cell death induced by the cytokine combination involved inflammatory cell death and pyroptosis. In addition, we found that complanatuside effectively alleviated inflammatory cell death in HaCaT cells induced by the cytokine combination. As far as we know, this is the first study reporting the combined effect of IFN-γ, TNF-α, and IL-6 on pyroptosis in skin keratinocytes and the protective effect of complanatuside on cytokine-induced pyroptosis in HaCaT cells.

Recent studies have indicated that IL-6, TNF-α, and IFN-γ were intensely elevated in COVID-19 patients and had become a significant feature of COVID-19 diseases deterioration (Diorio et al., 2020; Lucas et al., 2020; Karki et al., 2021). A recent study demonstrated that IFN-γ combined with TNF-α had a strong effect on inducing inflammatory cell death (Karki et al., 2021). Since IL-6 was found elevated in COVID-19, it is of great interest to explore the combined effect of IFN-γ, TNF-α, with IL-6 on inflammatory cell damage. We found that the cytokine combination strongly decreased cell viability and increased cell death in HaCaT cells. Utilizing caspase-1 and IL-1β as markers, we found that the cytokine combination induced pyroptosis in HaCaT cells. Caspase-1 activation contributes to the maturation of cytokines and membrane pore forming protein gasdermine during the process of pyroptosis and can be used as a marker of pyroptosis. The proinflammatory cytokine IL-1β is released by the host cells and is involved in the process of pyroptosis. Results of our study demonstrated that the combination of IFN-γ, TNF-α, and IL-6 strongly increased caspase-1 activation and elevated the level of IL-1β, indicating that this cytokine combination induced pyroptosis in HaCaT cells.

In our continuing search for effective anti-inflammatory agents, a series of natural compounds were screened for their potential effect on alleviating cytokine-induced inflammatory response in skin keratinocytes. We found that complanatuside had protective effect on HaCaT cells treated with IFN-γ, TNF-α and IL-6. Complanatuside is a flavonol glycoside isolated from *Astragalus complanatus*, which is commonly used in traditional Chinese medicine clinics. Studies have shown that flavonoids from *Astragalus complanatus* exert anti-inflammatory, anticancer, and immuno-modulating activities (Ng et al., 2014; Li et al., 2018; Yao et al., 2018). However, the anti-inflammatory effect and the protective effect of complanatuside on skin keratinocytes have not been reported. In our study, we found that complanatuside alleviated the effect of the cytokine combination on decreasing HaCaT cell viability. We also found that complanatuside decreased HaCaT cell pyroptosis induced by the cytokine combination. These results indicate that complanatuside protect HaCaT cells from cytokine-induced inflammatory cell damage.

To further investigate the mechanisms underlying the protective effect of complanatuside, we determined the effects of complanatuside on the NOD-like receptor, protein 3 (NLRP3), apoptosis-associated speck-like protein containing a caspase recruitment domain (ASC), and gasdermin D (GSDMD). Studies have shown that pyroptosis in COVID-19 may be associated with activation of NLRP3, ASC, and GSDMD (Song et al., 2020). The NLRP3 inflammasome is a complex platform involved in the activation of caspase-1 and the maturation of IL-1β in pyroptosis. NLRP3 activation elevates the release of the inflammatory cytokine IL-1β and induces pyroptosis (Wang et al., 2019). Downstream of NLRP3, ASC plays a crucial role in the formation of inflammasomes and the recruitment of caspase-1 (Satoh et al., 2013). GSDMD is a crucial component of the NLRP3 inflammasome and is required for IL-1β secretion and pyroptosis (He et al., 2015). Our study showed that pyroptosis in HaCaT cells resulted from the treatment with IFN-γ, TNF-α, and IL-6 and was associated with the increase in NLRP3, ASC, and GSDMD, and treatment with complanatuside strongly decreased the levels of these proteins. The results of the present study suggest that the combination of IFN-γ, TNF-α, and IL-6 induces pyroptosis through the NLRP3 inflammasome pathway, and inhibition of this pathway by complanatuside can effectively reduce pyroptosis in skin keratinocytes.

The mechanisms for activation of the NLRP3 inflammasome are not entirely clear. Inducible nitric oxide synthase (iNOS), one of the important inflammation regulators, has recently been found to be involved in NLRP3 activation and pyroptosis (Cinelli et al., 2020; Liao et al., 2021). COX-2 is an inflammatory mediator factor, which plays an important role in inducing inflammation-related diseases (Kundu et al., 2018). It was shown that COX-2 enhanced the activation of NLRP3 inflammasome and pyroptosis (Hua et al., 2015). Results of our study showed that treatment with complanatuside resulted in decreases in the levels of iNOS and COX-2 in HaCaT cells treated with the combination of IFN-γ, TNF-α, and IL-6. This result supports the notion that the effect of complanatuside on inhibiting NLRP3 inflammasome is partly mediated via inhibition of iNOS and COX-2.

ROS is a second messenger that regulates the inflammatory response. Inflammation can increase the level of ROS, and overexpression of ROS can also aggravate the inflammatory response (Forrester et al., 2018). ROS is involved in the inflammatory response in COVID-19 (Morris et al., 2020) and activation of NLRP3 (Abais et al., 2015). In our study, we found that that the elevated level of ROS in HaCaT cells treated with the combination of IFN-γ, TNF-α, and IL-6 was strongly reduced upon treatment with complanatuside. This result, together with the inhibitory effect of complanatuside on iNOS and COX-2 as described above, suggests that multiple mechanisms may contribute to the protective effect of complanatuside on cytokine-induced pyroptosis. Simultaneously inhibition of iNOS, COX-2, and ROS may be an effective strategy for alleviating cytokine storm induced skin lesion in COVID-19 patients.

## Conclusion

To sum up, our results demonstrated that complanatuside alleviated inflammatory cell damage induced by the IFN-γ, TNF-α, and IL-6 combination in skin keratinocytes. Complanatuside protected HaCaT cells from cytokine-induced pyroptosis as evidenced by decreased caspase-1 and IL-1β. Mechanistic studies showed that the pyroptosis related NLRP3 pathway was inhibited by complanatuside. The suppression of NLRP3 activation and pyroptosis was associated with inhibition of iNOS, COX-2, and ROS. Complanatuside may be a promising candidate for inhibiting COVID-19 related skin inflammatory damage.

## Data Availability

The original contributions presented in the study are included in the article/Supplementary Material, further inquiries can be directed to the corresponding authors.

## Abbreviations

COVID-19, Coronavirus disease-19; IL, interleukin; TNF, tumor necrosis factor; IFN, interferon; ROS, reactive oxygen species; DMEM, Dulbecco’s Modified Eagle’s Medium; FBS, Fetal bovine serum; MTT, 3-(4,5-Dimethythiazol-2-yl)-2,5-diphenyl-tetra-zoliumbromide; iNOS, inducible nitric oxide synthase; COX-2, cyclooxygenase-2; NLRP3, NOD-like receptors protein 3; ASC, apoptosis-associated speck-like protein containing a caspase recruitment domain; GSDMD, gasdermin D; SDS-PAGE, sodium dodecyl sulfate polyacrylamide gel electrophoresis.
